# Effects of Different Metals on Photosynthesis: Cadmium and Zinc Affect Chlorophyll Fluorescence in Durum Wheat

**DOI:** 10.3390/ijms19030787

**Published:** 2018-03-09

**Authors:** Momchil Paunov, Lyubka Koleva, Andon Vassilev, Jaco Vangronsveld, Vasilij Goltsev

**Affiliations:** 1 Department of Biophysics and Radiobiology, Faculty of Biology, Sofia University, 1164 Sofia, Bulgaria; mokavey@abv.bg (M.P.); goltsev@biofac.uni-sofia.bg (V.G.); 2 Department of Plant Physiology and Biochemistry, Agricultural University, 4000 Plovdiv, Bulgaria; l_koleva2001@yahoo.com; 3 Centre for Environmental Sciences, Hasselt University, 3590 Diepenbeek, Belgium; jaco.vangronsveld@uhasselt.be

**Keywords:** photosynthesis, chlorophyll fluorescence, cadmium, zinc

## Abstract

A comparative study of the effects of exposure to high Cd^2+^ (50 µM) and excess Zn^2+^ (600 µM) on photosynthetic performance of hydroponically-grown durum wheat seedlings was performed. At day 8, Cd and Zn were added to the nutrient solution. After 7-days exposure, the chosen concentrations of both metals resulted in similar relative growth rate (RGR) inhibitions of about 50% and comparable retardations of the CO_2_ assimilation rates (about 30%) in the second developed leaf of wheat seedlings. Analysis of chlorophyll a fluorescence indicated that both metals disturbed photosynthetic electron transport processes which led to a 4- to 5-fold suppression of the efficiency of energy transformation in Photosystem II. Non-specific toxic effects of Cd and Zn, which prevailed, were an inactivation of part of Photosystem II reaction centres and their transformation into excitation quenching forms as well as disturbed electron transport in the oxygen-evolving complex. The specificity of the Cd and Zn modes of action was mainly expressed in the intensity of the toxicity effects: despite the similar inhibitions of the CO_2_ assimilation rates, the wheat photochemistry showed much more sensitivity to Cd than to Zn exposure.

## 1. Introduction

Metal contamination is one of the most important environmental problems. It is mostly due to industrial and agricultural activities, such as smelter and incinerator emissions, traffic, dispersal of mining wastes, use of contaminated sewage sludges, manures, phosphate fertilizers, etc. [1,2]. Cadmium (Cd) and zinc (Zn) are among the most problematic metals, creating potential risks for both plant production and animal and human health.

Most studies on plant–metal interactions are focused on the processes involved in metal ion uptake and accumulation in the harvestable crop parts as well as on metal-induced phytotoxicity. Cadmium has no known biological functions, but is easily taken up by plants [3,4]. From plants, it may accumulate into food chains, where it may, for example, cause harmful effects on human health. Yet there exist a few reports demonstrating a possible biological role of Cd in plants: as a substitute for Zn in the Zn/Cd hyperaccumulator *Sedum alfredii* [5] as well as a feeding deterrent against *Frankliniella occidentalis* (thrips) in the hyperaccumulator *Thlaspi caerulescens* ([6] and references therein). Cadmium may also provoke significant phytotoxic effects even in relatively low tissue concentrations (micro-element range). Different from Cd, Zn is an essential element for all plants, but excess Zn induces functional disorders in plants. The phytotoxicity threshold for Zn concentrations in plant tissues are in the range of 200–500 mg kg^−1^ [7,8].

The visible toxicity symptoms in Cd- and Zn-exposed plants are similar: inhibited germination, stunted growth, leaf chlorosis, necrotic spots, etc. [9,10]. The described toxic effects of both metals on cardinal physiological processes in plants, such as photosynthesis, water relations, mineral nutrition, dark respiration, etc. are also comparable [11,12,13,14,15,16]. For example, it has been shown that both metals can induce similar or identical disturbances of photosynthesis at different structural-functional levels: pigments and light capture, thylakoid ultrastructure and photosynthetic electron transport, stomatal conductance and access of CO_2_, activities of Calvin cycle enzymes, etc. [11,17,18,19].

Cadmium and Zn-induced effects in the light-dependent photosynthetic processes have been studied in both in vitro and in vivo conditions [20,21,22,23]. In in vitro studies, it was established that both metals can significantly decrease the activities of photosystem II (PSII) and, to lesser extent, also of photosystem I (PSI) as well as the rate of photosynthetic electron transport [17,24]. However, in vivo studies obtained by chlorophyll *a* fluorescence (ChlF) techniques repeatedly reported that the photochemical reactions are not so sensitive to Cd and Zn than Calvin’s cycle reactions [19,25,26]. Partially, this inconsistency could be explained by the fact that in vitro studies are performed without substrate limitation and occurrence of feedback inhibition, while in vivo the metal-induced alterations in primary C metabolism may lead to a down-regulation of PSII activity [21,25,27]. Hence, the effects of high Cd and excess Zn concentrations on the light-dependent photosynthetic processes are still not fully understood.

The most common and widely used ChlF analyses are performed on dark- and light-adapted leaf samples, and subsequently different parameters characterizing the steady-state status of the photosynthetic apparatus are calculated [28]. More recently, the so-called JIP-test was introduced in chlorophyll fluorescence analyses [29]. The JIP-test is based on measurements of ChlF fast kinetics and on analysis of signals providing detailed information on the structure and function of the photosynthetic apparatus (PSA), primarily PSII. The models underlying the JIP-test describe the primary photosynthetic reactions by taking into account the structure of PSA in full consistency with the theory of energy fluxes occurring in the thylakoid membrane between the complexes of photosynthetic pigments in PSII [30,31]. In the past few years, this test was widely used for plant performance testing under stressful conditions [31,32,33,34]. For example, it has been applied for the evaluation of plant performance under conditions of light [35], chilling [36,37], high temperature [38,39,40], and drought [41,42,43,44] stress. More recently, the JIP test was also applied to evaluate the photosynthetic performance of plants exposed to different metals [45,46,47,48]. Unfortunately, until now, the JIP-test information published for plants exposed to excess Zn is very scant. The available JIP-test information for Cd-exposed maize plants was obtained in conditions that did not induce growth inhibition [48].

Despite the relatively rich dataset concerning effects of high Cd and excess Zn concentrations on plant performance, it is obvious that there are still many open questions. For example, it is still not clear enough whether these metals may cause specific effects on photosynthesis or not. Considering the fact that the biological functions of these metals are completely different, it is reasonable to expect their impact on plant performance also to differ. Unfortunately, comparative studies on Cd- and Zn-induced phytotoxicity effects are scarce. Considering the lack of sufficient and detailed information concerning in vivo measured effects of Cd and Zn on primary photosynthetic processes, we decided to perform a comparative study. To compare the phytotoxic effects of non-essential (Cd) and essential (Zn) elements, our experimental design consisted of exposing plants to external Cd and Zn concentrations that produced identical inhibition of plant growth.

## 2. Results

The wheat seedlings exposed to 50 µM Cd^2+^ and 600 µM Zn^2+^ in the medium manifested clear toxicity symptoms, such as the appearance of chlorotic and necrotic leaf spots, weaker development of side roots, and root browning [2,10]. There were some differences in the visible toxicity symptoms in the leaves of Zn- and Cd-exposed plants. While Zn caused necrotic spots, Cd induced chlorosis. Exposure to Cd resulted in browning and a stronger inhibition of root length growth and branching, whereas Zn exposure lead to roots that were lighter in color and thinner in diameter. 

Dry weight increase and RGR of Cd- and Zn-exposed wheat seedlings were inhibited by approximately 50%, while their net photosynthetic rate (A) was about 35% lower (Table 1). The mineral analysis revealed that both Cd and Zn concentrations in the roots of exposed plants (936 mg Cd kg^−1^ dry weight and 3029 mg Zn kg^−1^ dry weight) were several-fold higher than those in the leaves (150 mg Cd kg^−1^ dry weight and 880 mg Zn kg^−1^ dry weight). Cadmium and Zn exposure strongly diminished chlorophyll and carotenoid concentrations (Table 2). The decreases of chlorophyll *a* concentrations due to Cd and Zn exposure were higher than 50%, while chlorophyll *b* was less affected. Cadmium tended to inhibit the chlorophyll b concentration a bit more than Zn (although not significant). The total carotenoid concentration was more inhibited by Zn.

The similar inhibitions of both RGR and A in Cd- and Zn-exposed wheat seedlings provided a good opportunity to compare the toxic effects of these metals more in detail.

**Figure 1:**
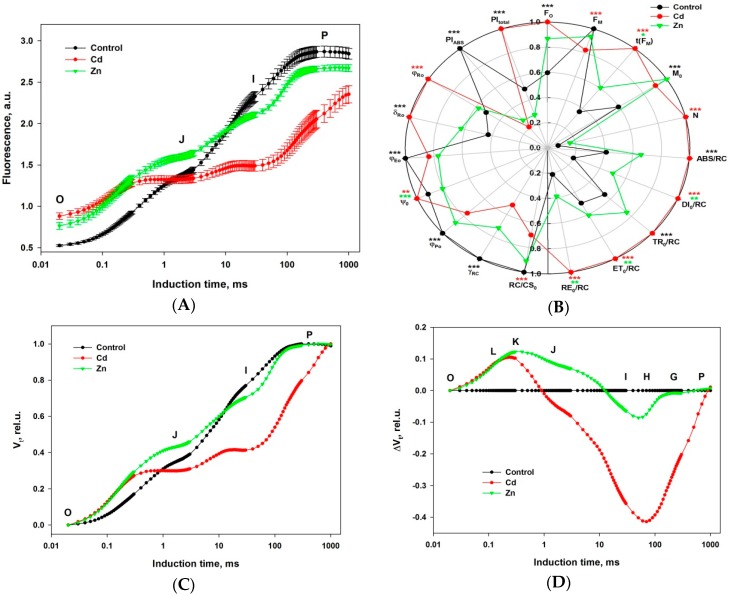
(**A**) Induction transients of chlorophyll *a* fluorescence in leaves of wheat (*Triticum durum* Desf. (cv. Beloslava)) plants—control (black), exposed to 50 µM Cd (red) and 600 µM Zn (green). The whole plants were adapted to dark in room for 1 h and additionally the measured spots were kept in dark in the clip for 1 min just before measurement. Induction curves of ChlF were recorded for 1 s with 3000 μmol m^−2^ s^−1^ PPFD. Each point in the graph is an averaged value of 6 repetitions and the standard errors are shown. The characteristic levels (O, J, I, and P) of the induction transients are denoted with letters. (**B**) Parameters of the JIP test (described in Table 1) are calculated from the curves shown in A. The asterisks above each parameter indicate if there is statistical significance for the Cd (red) or Zn exposure (green) or for both treatments (black): 1 asterisk—*p* < 0.05, 2—*p* ≤ 0.01, and 3—*p* ≤ 0.001. (**C**) Induction curves of the relative variable chlorophyll fluorescence (V_t_) resulting from double normalization of the values in (**A**) to the minimal (F_O_) and maximal (F_M_) levels. The characteristic levels (O, J, I, and P) of the induction curves are denoted as in (**A**). (**D**) Curves of differential values (ΔV_t_) resulting from subtracting the control from exposed V_t_. The times of the induction curves characteristic levels (O, J, I, and P) are marked as well as the intermediary levels (L, K, H, and G) at which ΔV_t_ peaks and shoulders occur.

The state of the light-dependent photosynthetic processes in Cd- and Zn-exposed wheat plants was analyzed applying the JIP test. This approach allows monitoring of molecular processes occurring at the level of the photosynthetic machinery during the light phase in leaves: energy migration between neighboring photosynthetic units and electron transfer reactions at donor and acceptor sites of both PSII and PSI and between them [49,50]. The induction curves of chlorophyll fluorescence recorded for 1 s in dark-adapted plants are shown in Figure 1A. The increase of the initial chlorophyll fluorescence level (F_O_), the decrease of the maximal level (F_M_), and the change of the transient’s shape are clearly visible in the exposed plants compared to the non-exposed control. All these effects show that the photosynthetic process is strongly affected by metal exposure. The F_O_ rise indicates decreased energy trapping efficiency of PSII. The reduced efficiency of the energy transfer from the antenna to the RC was demonstrated by Havaux [51] and Yamane et al. [52] for heat-stressed photosynthesizing leaves. The former could be due to the metals disturbing the structure of the RC complexes and the latter because of swelling of the thylakoid membranes due to hygroscopic or charge shielding action of the divalent metal ions leading to a pulling apart of the complexes, lowering the energy transfer efficiency. The measured maximal level of chlorophyll fluorescence (F_M_) is a complex parameter that is dependent on the structural leaf tissue characteristics determining actinic light absorption, reflection, and reabsorption of emitted chlorophyll fluorescence including the chlorophyll content in the leaf (see [31] and the references therein). The F_M_ decline could be partly related to a reduced chlorophyll *a* concentration which is observed in metal exposed plants (Table 2). The presence of chlorophyll fluorescence quenchers could lower F_M_ as well. Such molecules could be endogenous like carotenoids or exogenous like oxygen. However, in exposed plants the carotenoid contents decreased. Although, it is well known that under strong stress the chlorophyll molecules become more easily accessible to oxygen and other exogenous quenchers. The assumptions made above are well supported by the values of the main JIP parameters (Figure 1B). Each parameter is explained in Table 3. There were statistically significant differences between Cd-exposed and non-exposed control plants for all parameters and for all but F_M_, N, RC/CS_0_, and φ_Ro_ parameters in the case of Zn-exposed plants. 

To better analyze the differences in the induction curve’s shape, the transients of the double normalized fluorescent signal (to F_O_ and F_M_) are presented in Figure 1C. In this way, the deviations in the intermediate induction phases of exposed plants with respect to the control become clear:

- steeper initial rise for both metals,
- increase for Zn, decrease for Cd of the V_J_ (2 ms), and
- decrease (very strong for Cd) of the V_I_ (30 ms).

Noteworthy is the fact that not only are the V_J_ and V_I_ levels are much lower for Cd-exposed seedlings, but that the rise before and after them is heavily slowed down with chlorophyll fluorescence still rising 1 s after the start of induction, probably not reaching the real maximal level (F_M_). This observation could be explained by a high availability of efficient chlorophyll fluorescence quenchers or formation of inactive PSII reaction centers with destroyed oxygen-evolving complex (OEC), that are able to quench a large part of excitation in PSII antennae complexes.

Differential curves resulting from subtracting the control from the Zn- and Cd-exposed double normalized fluorescent values are presented in Figure 1D. Thus the 3 bullet points outlined above can be visualized as:

- positive peak in F_O_-F_J_,
- shoulder in F_J_-F_I_ and
- negative peak in F_I_-F_P_ transient.

The differential curves constructed in the first and the second time intervals should be connected with excitation energy redistribution between neighboring PSII antenna complexes as well as with the balance of electron transport reactions around PSII. The increased level at 0.3 ms, called the (positive) K-band, is a typical characteristic in the case of high temperature stress, indicating a reduced rate of electron donation to RC due to impaired OEC [53]. On the other side, a K-band could be connected to a higher rate of electron transport from RC to its acceptors. In conclusion, a positive K-band occurs whenever the balance of electron transport around PSII is causing electron deficit and formation of P_680_^+^ that is an effective quencher of excited state of chlorophyll molecules [53,54]. That molecule is in fact a powerful chlorophyll fluorescence quencher—absorbed light cannot be utilized in photochemistry and dissipates as heat. High levels of P_680_^+^ may contribute to the striking quenching observed in Cd exposed seedlings. 

The increased F_J_ level and the shoulder in the F_J_-F_I_ transient in the case of Zn exposure could be connected with a reduced rate of electron transfer from Q_A_ to Q_B_ at the acceptor side of PSII. These effects as well as the positive K-band should lower the photosynthetic electron flow from PSII to PSI. The decreased F_J_ level, despite the positive peak in the initial rise of F_O_-F_J_ in the case of Cd exposure, suggests vast quantities of P_680_^+^ and/or highly effective quenching by exogenous molecules. 

The decreased F_I_ level and the negative peak in the F_I_-F_P_ transient could be connected with a bigger pool of intersystem (plastoquinone) and/or PSI end electron acceptors per active PSII RC in the exposed plants compared to the control. 

**Figure 2:**
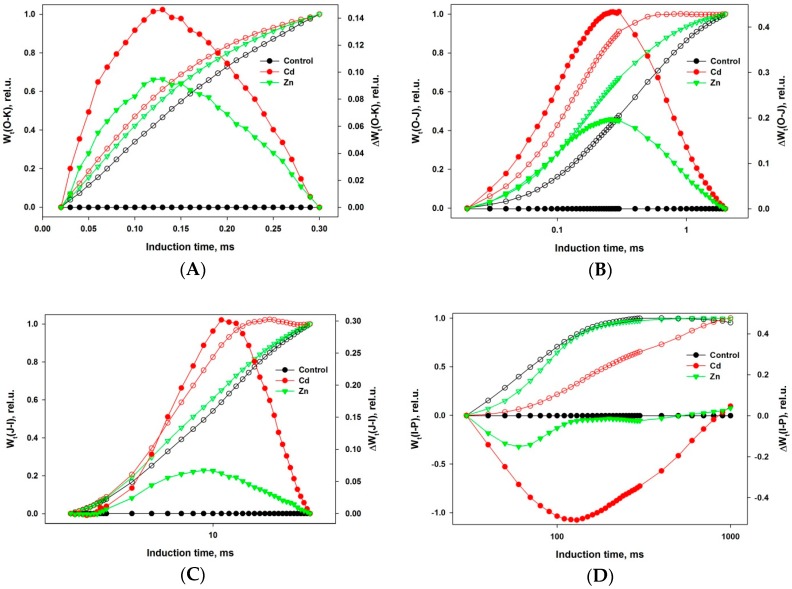
Combined graphs of differential curves (filled circles) and their corresponding double normalized chlorophyll fluorescence signal (empty circles): (**A**) from O to K (L band), (**B**) from O to J (K band), (**C**) from J to I (H), and (**D**) from I to P (G band). All these transients are constructed from the corresponding values of the curves shown in Figure 1A and the series designation is the same: control plants (black), exposed to Cd (red), and to Zn (green). There is a statistically significant difference for the maximal values of the differential curves of exposed plants compared to the control with *p* < 0.05.

All the observations described above can be demonstrated more precisely by constructing differential curves within different parts of the induction. Such curves are plotted on Figure 2A–D for 4 different time intervals, representing formation of bands: L (interval O-K), K (O-J), H (J-I), and G (I-P). 

Positive L, K, and H bands are seen in Figure 2A–C as well as a negative G band (Figure 2D) for both metal exposures. However, the effects of Cd exposure are much more obvious in every case. Interestingly, the times for the extreme of each band are the same for both metals for L (120 µs) and K (250 µs) but they differ slightly for H (Zn—9 ms, Cd—11 ms) and significantly for G (Zn—60 ms, Cd—130 ms), demonstrating that the specificity of the Cd and Zn effects is exhibited in electron transport after PSII. 

## 3. Discussion

To describe the responses of the wheat photosynthetic apparatus to Cd- and Zn-exposure, we used the analysis of the photoinduced induction transients of chlorophyll fluorescence [44,49,55]. The dynamics of the chlorophyll fluorescence rise are directly related to redox reactions not only in PSII, but throughout the entire photosynthetic electron transport chain. The application of the JIP test [56] allows analysis of the changes in important energetic characteristics of the PSA as well as the stress-induced processes in the light phase of photosynthesis under in vivo conditions (Table 3).

Metal exposure disturbs the energy migration from the antenna complexes to the chlorophyll of the reaction centers which leads to an increased chlorophyll fluorescence emission in dark-adapted objects (F_O_). In this study, Cd exhibited a much more effective inhibition than Zn (see Figure 1A). One of the effects is the inactivation of a part of the reaction centers of PSII, which are converted in so-called “energy sinks”, thus transforming the excitation energy of the antenna chlorophylls into heat energy. This is demonstrated by the parameters γ_RC_ (representing the relative part of photochemically active molecules, P_680_, in the total pool of chlorophyll molecules in PSII) and RC/CS_0_ (representing the relative concentration of active RC per unit illuminated area). A consequence of this effect is also the changes of the relative antenna size linked to each active RC (represented by the parameter ABS/RC), which increases in metal-exposed wheat plants, particularly in the case of Cd-exposure. The reduced number of active RC leads to an increase of necessary numbers of RC turnovers for full reduction of the plastoquinone pool (parameter N). Accordingly, a certain suppression of the RC photochemical efficiency is detected, presented by the quantum yield of the primary photochemical reaction (φP_0_), while the re-oxidation reaction of the primary quinone acceptor Q_A_^−^ is slightly reduced by Zn-exposure and even probably activated in case of Cd-exposure [57].

Generally, the dynamics of the induction transient may be successfully analyzed by plotting of differential curves [56] (see Figure 1D). The stress factors strongly modify the shapes of the differential curves and the effects are manifested in different ways, forming bands of the induction transient. Strasser et al. [56] suggested nomenclature for the designation of different transient phases by the letters (bands) L, K, J, I, H, and G. 

For a more detailed characterization of the impact of metal exposure on the PSA of the wheat seedlings, we analyzed the stress-induced changes of the shape of the fast chlorophyll fluorescence rise from the O to P phase (Figure 1D) as well as separate sections of the this integral transient in the time course intervals: 20–300 µs, 20–2000 µs, 2–30 ms, and 30–300 ms.

The shape of the induction curve between 20 and 300 µs (usually called the L band) is influenced by the excitation energy transfer between PSII units, commonly denoted as “connectivity” or “grouping” [58,59].

The appearance of a positive L band (Figure 2A) indicates a weaker connectivity between adjacent PSIIs at the level of the antenna complexes. This means that Zn and Cd also affect the structure of the thylakoid membranes, expressed by a decreased transfer of excitation energy between adjacent photosynthetic units [60]. The changes of the energetic interactions could be a result of stacking/destacking of thylakoid membranes caused by electrostatic interaction at different ion concentrations [61,62]. 

The K band occurring during the O-J transient (Figure 2B) is usually explained as a result of an imbalance in electron transfer reactions at donor and acceptor sides of PSII [53]. The appearance of a positive K-band is usually explained as a consequence of a retardation of electron donation from the OEC to oxidized chlorophyll, leading at light to the formation of an increased P680^+^ concentration able to effectively quench the excited antenna chlorophylls. The maximal chlorophyll fluorescence level of the induction curve, reflecting the state of PSA with fully closed PSII RCs, is significantly decreased in Cd-exposed wheat seedlings as compared to the control and even to Zn-exposed ones (see point P in Figure 1А and F_M_ in Figure 1B). The appearance of a K band was also observed at high temperature exposure of PSA and may be due to dissociation of the PsbO protein (Mn stabilizing protein) from the OEC [63,64]. The strongly expressed K-band demonstrated that both metals, but especially Cd, disturbed the functioning of OEC in PSII. 

The chlorophyll fluorescence rise within the thermal phase of the induction kinetics is rather complex and depends on different factors, which were described in detail in a comprehensive review [50]. A more simplified model describes the induction transient phases by energy fluxes at different stages of the photosynthetic light phase [49]. In the frame of this model, the photoinduced transient from phase J (2 ms) to I (30 ms) represents the dynamics of the plastoquinone pool reduction between both photosystems. Phase I is formed when a dynamic equilibrium (quasi-steady-state condition) is reached between reduction of the plastoquinone pool by the electron flow originating from PSII and its re-oxidation due to PSI activity. The comparison of the transient dynamics between control (non-exposed) and metal-exposed plants in that time interval indicates that Cd and Zn ions influenced both redox reactions. The visible positive band (especially in case of Cd-exposure) (Figure 2C) could reflect a decrease in the relative numbers of active plastoquinone molecules reduced by each active RC of PSII. 

During the time interval for the induction transient from I to P, the dynamics are defined by the reduction speed of the terminal electron acceptors in PSI. It could be suggested that the strongly expressed negative bands (see Figure 2D) indicate an increase of the pool of the potential electron acceptors and a general decrease of the linear electron flux through both photosystems. 

In conclusion, both Cd and Zn had clear negative effects on the plant processes considered in this experiment. However, we found that these metals exhibit specificity concerning their effects on PSA of wheat plants in circumstances under which the effects on integral processes were very similar (parameters DW, RGR, A, and chlorophyll content). This specificity was expressed in the intensity of the effect: PSA was much more sensitive to 50 μM Cd than to 600 μM Zn exposure.

## 4. Materials and Methods

Seeds of *Triticum durum* Desf. (cv. Beloslava) were germinated on wet filter paper and the seedlings were transferred to pots filled with nutrient solution with pH = 5.8 ± 0.1 containing: 0.505 mM KNO_3_, 0.15 mM Ca(NO_3_)_2_ × 4H_2_O, 0.1 mM NH_4_H_2_PO_4_, 0.1 mM MgSO_4_ × 7H_2_O, 4.63 mM H_3_BO_3_, 0.91 mM MnCl_2_ × 4H_2_O, 0.03 mM CuSO_4_ × 5H_2_O, 0.06 mM H_2_MoO_4_ × H_2_O, 0.16 mM ZnSO_4_ × 7H_2_O, 1.64 mM FeSO_4_ × 7H_2_O, and 0.81 mM Na_2_–EDTA. Nutrient solution was refreshed every other day and aerated continuously. The seedlings were grown in a growth chamber under controlled environmental conditions: photoperiod 16/8 h (light/dark), 250 μmol m^−2^ s^−1^ photosynthetic photon flux density (PPFD), 26/22 °C day/night temperature and 60–65% relative air humidity. Nutrient solutions were refreshed every other day and aerated continuously. When plants were 8 days old, they were exposed to the high Cd and Zn concentrations in the nutrient solution for 7 days. The experimental design included 3 treatments: (1) unexposed seedlings (control), (2) seedlings exposed to 50 μM Cd^2+^ (3CdSO_4_ × 8H_2_O) and (3) seedlings exposed to 600 μM Zn^2+^ (ZnSO_4_ × 7H_2_O). These concentrations were chosen based on preliminary dose-response experiments. At the end of experimental period, wheat plants were used for different analyses.

Dry weight of the plants was determined after drying them for 24 h at 65 °C. Relative growth rates (RGR) of the plants from different treatments were calculated according to Beadle [65]. RGR = (lnDW_2_ − lnDW_1_)/t, where DW_2_ and DW_1_ stand for final and initial weights of the plants and t − experimental period (7 days).

Total contents of Cd and Zn in roots and leaves were determined by inductively coupled plasma-atomic emission spectroscopy (ICP-AES) after dry mineralization at 500 °C preceded by HNO_3_ treatment. 

Net photosynthetic rate (A) was measured on the second developed leaf of the plants with an open photosynthetic system LCA-4 (Analytical Development Company Ltd., Hoddesdon, UK), equipped with a narrow chamber at the experimental conditions described above. 

Photosynthetic pigments (chlorophyll *a*, chlorophyll *b* and total carotenoids) were extracted in 80% acetone, determined spectrophotometrically, and calculated according to the formulae of Lichtenthaler [66].

Chlorophyll fluorescence analysis was performed using a Handy PEA fluorimeter (Handy Plant Efficiency Analyzer, Hansatech Instruments Ltd., King’s Lynn, UK) on native leaves of plants 7 days after start of the treatment (DAT). The whole plants were adapted to darkness in room for 1 h and additionally the measured spots were kept in darkness in the clip for 1 min just before measurement. Induction curves of ChlF were recorded for 1 s with 3000 μmol m^−2^ s^−1^ PPFD. For each experimental treatment, at least 10 measurements were performed. The primary data processing was done using the HandyBarley program, developed by Petko Chernev at the Department of Biophysics and Radiobiology, Faculty of Biology, Sofia University, and the secondary processing, including calculation of JIP parameters—on Microsoft Excel. The plots were made in Sigma Plot.

The intensity of the ChlF was recorded in arbitrary units. Those were transformed into relative units of the relative variable chlorophyll fluorescence (V_t_) by double normalization to the initial, minimum level, F_O_, and to the maximum level, F_M_. When the V_t_ values of the untreated control were subtracted from the values of the other treatments at the corresponding moment in the induction time, the differential curves were built. For more detailed analysis of the processes occurring within the induction time, the chlorophyll fluorescence rise was analyzed within 4 time intervals and differential curves were constructed for each of them. Such curves were made by double normalization to: F_O_ and F_J_ (level at 2 ms), F_O_ and F_K_ (at 0.3 ms), F_J_ and F_I_ (at 30 ms) and F_I_ and F_P_. 

The chlorophyll fluorescence intensity values determined at 50 μs, 100 μs, and 300 μs, along with F_O_, F_J_, F_I_, and F_M_ were used for the calculation of the OJIP test parameters [49,55,56,58,67], which are presented in Table 3.

Statistical analysis of physiological parameters was performed using a one-way ANOVA (for *p* < 0.05). Based on ANOVA results, a Tukey’s test for main comparison at a 95% confidential level was applied. Statistical analysis for ChlF parameters was performed with the program Sigma Plot version 11 using one-way ANOVA and the Holm–Sidak method for multiple comparisons with overall significance level of 0.05. 

## Figures and Tables

**Table 1:** Effects of Cd and Zn on growth parameters (DW—fresh weight, RGR—relative growth rate) and net photosynthetic rate (A) of durum wheat plants.

Treatments	Parameters					
	DW (mg plant^−1^) Inhibition %		RGR (mg g DW^−1^ day^−1^) Inhibition %		A (µmol CO_2_ m^−2^ s^−1^) Inhibition %	
Control	484 a	0	140.6 a	0	6.02 a	0
Cd 50 µM	271 b	55	68.8 b	51	3.94 b	35
Zn 600 µM	252 b	58	64.1 b	53	4.02 b	33

Values followed by different letters (a, b) within a column are significantly different at *p* < 0.05.

**Table 2:** Effects of Cd and Zn on photosynthetic pigments content in durum wheat plants.

Treatments	Photosynthetic Pigments Content (mg g FW^−1^)					
	Chlorophyll *a*	Inhibition %	Chlorophyll *b*	Inhibition %	Carotenoids	Inhibition %
Control	1.77 a	0	0.70 a	0	0.47 a	0
Cd 50 µM	0.85 b	52	0.48 b	31	0.32 b	32
Zn 600 µM	0.80 b	55	0.53 b	24	0.26 c	45

Values followed by different letters (a, b, c) within a column are significantly different at *p* < 0.05.

**Table 3:** Definitions of measured and calculated chlorophyll *a* fluorescence parameters used in the experiment (Based on [49,55,56,58,67]).

Chlorophyll Fluorescence Parameter	Description
Measured parameters and basic JIP-test parameters derived from the OJIP transient	
F_O_ ~ F_20µs_	Minimum fluorescence, when all PSII reaction centers (RCs) are open; Fluorescence intensity at 20 µs
F_J_	Fluorescence at the J-step (2 ms) of the O-J-I-P transient
F_I_	Fluorescence at the I-step (30 ms) of the O-J-I-P transient
F_M_ = F_P_	Maximum recorded fluorescence at the P-step when all RCs are closed
t(F_M_)	Time (in ms) to reach maximal fluorescence F_M_
M_0_ = 4 × [(F_300µs_ − F_50µs_)/(F_M_ − F_50µs_)]	Approximated initial slope (in ms^−1^) of the fluorescent transient. This parameter is related to the rate of closure of reaction centers
N = S_m_/S_s_ = S_m_ × M_0_ × (1/V_J_)	Turnover number: number of Q_A_ reduction events between t = 0 and t(F_M_), where S_m_ is normalized total complementary area above the O-J-I-P transient (reflecting multiple-turnover Q_A_ reduction events), S_s_—normalized total complementary area corresponding only to the O-J phase (reflecting single-turnover Q_A_ reduction events) and V_J_—relative variable fluorescence at the J-step
Specific energy fluxes (per active, i.e., Q_A_-reducing PSII RC)	
ABS/RC = M_0_ × (1/V_J_) × (1/φ_Po_)	Absorption flux per RC corresponding directly to its apparent antenna size—ratio between chlorophyll in antenna and chlorophyll in RC
DI_0_/RC = (ABS/RC) − (TR_0_/RC)	Dissipated energy flux per RC at the initial moment of the measurement, i.e., at t = 0
TR_0_/RC = M_0_ × (1/V_J_)	Trapping flux leading to Q_A_ reduction per RC at t = 0
ET_0_/RC = M_0_ × (1/V_J_) × (1 − V_J_)	Electron transport flux from Q_A_^−^ to plastoquinone per RC at t = 0
RE_0_/RC = M_0_ × (1/V_J_) × (1 − V_I_)	Electron transport flux from Q_A_^−^ to the PSI end electron acceptors per RC at t = 0, where V_I_ is the relative variable fluorescence at the I-step
Density of reaction centers	
RC/CS_0_ = φ_Po_ × (V_J_/M_0_) × F_O_	Density of active PSII RCs. CS denotes cross section
Quantum yields and probabilities	
γ_RC_ = 1/[(ABS/RC) + 1] = RC/(ABS + RC)	Probability that PSII chlorophyll molecule functions as RC
φ_Po_ = TR_0_/ABS = [1 − (F_O_/F_M_)]	Maximum quantum yield of primary PSII photochemistry (at t = 0)
ψ_0_ = ET_0_/TR_0_ = 1 − V_J_	Probability (at t = 0) that a trapped exciton moves an electron into the electron transport chain beyond Q_A_^−^
φ_Eo_ = ET_0_/ABS = φ_Po_ × ψ_Eo_	Quantum yield (at t = 0) for electron transport from Q_A_^−^ to plastoquinone
δ_Ro_ = RE_0_/ET_0_ = (1 − V_I_)/(1 − V_J_)	Efficiency/probability (at t = 0) with which an electron from the intersystem carriers moves to reduce end electron acceptors at the PSI acceptor side
φ_Ro_ = RE_0_/ABS = φ_Eo_ × δ_Ro_	Quantum yield (at t = 0) for reduction of end electron acceptors at the PSI acceptor side
Performance indexes	
PI_ABS_ = γ_RC_/(1 − γ_RC_) × φ_Po_/(1 − φ_Po_) × ψ_Eo_/(1 − ψ_Eo_)	Performance index of PSII based on absorption
PI_total_ = PI_ABS_ × δ_Ro_/(1 − δ_Ro_)	Performance index of electron flux to the final PSI electron acceptors, i.e., of both PSII and PSI
